# From EEG Source Localization to Brain Network Analysis: A Computational Neuroscience Perspective

**DOI:** 10.3390/biology15181622

**Published:** 2026-09-15

**Authors:** Jooyoung Lee, Tae-Hoon Eom

**Affiliations:** Department of Pediatrics, College of Medicine, The Catholic University of Korea, Seoul 06591, Republic of Korea; jy37jy@naver.com

**Keywords:** EEG source localization, EEG source imaging, forward modeling, inverse problem, functional connectivity, effective connectivity, brain network analysis, graph theory, machine learning, deep learning

## Abstract

Electroencephalography (EEG) source localization has evolved from a technical solution to an inverse problem and into a fundamental framework for investigating large-scale brain networks. By reconstructing cortical activity from scalp-recorded signals, source-space EEG improves anatomical interpretability and reduces confounding effects of volume conduction compared with sensor-level analyses. These advances have enabled more reliable estimation of functional and effective connectivity, graph-theoretical characterization of brain networks, and the integration of machine learning for automated analysis and clinical decision support. Nevertheless, the accuracy and reproducibility of source-level analyses remain highly dependent on forward modeling, inverse algorithms, preprocessing strategies, and connectivity estimation methods. Recent methodological developments have further expanded the role of EEG source imaging in computational neuroscience, supporting applications ranging from network-based investigations of brain function to biomarker discovery and disease classification. This review summarizes the computational framework underlying source-level EEG, beginning with the forward and inverse problems and progressing through source reconstruction, connectivity analysis, graph-theoretical network modeling, and emerging machine learning and deep learning techniques. We also highlight current clinical applications and major methodological challenges, including source leakage, model uncertainty, reproducibility, and interpretability, and outline future directions for robust and clinically translatable source-level EEG analysis.

## Simple Summary

Electroencephalography is a safe and noninvasive technique for recording the brain’s electrical activity that has long been used to study brain function and diagnose neurological disorders. Recent advances now allow researchers to estimate where the electrical signals originate inside the brain and examine how different brain regions communicate as networks, rather than as isolated areas, developments that provide new opportunities to better understand brain function, improve the diagnosis and treatment of disorders such as epilepsy, and identify reliable markers of disease. This review outlines the principles of brain signal localization, methods for analyzing communication among brain regions, and approaches for studying the organization of brain networks, in addition to discussing how artificial intelligence can help analyze these complex data while emphasizing the importance of accurate modeling, standardized analysis methods, and careful validation. By investigating these rapidly developing fields together, this review highlights current achievements, remaining challenges, and future directions for development. These advances have the potential to improve both neuroscience research and clinical care by providing more accurate and personalized tools for understanding brain disorders.

## 1. Introduction

Electroencephalography (EEG) is a widely used, noninvasive technique for investigating human brain function that provides millisecond temporal resolution and has broad clinical and research applicability [1,2]. Scalp-recorded EEG reflects a spatially blurred mixture of underlying neural generators; its interpretation therefore requires solving an ill-posed inverse problem through explicit forward modeling and source reconstruction [1,2,3]. Accurate EEG source localization depends on realistic head models, appropriate inverse algorithms, and careful consideration of the assumptions linking measured potentials to cortical activity [2,3,4,5].

EEG source localization has developed from a purely technical inverse problem to a methodological bridge between sensor-level recordings and system-level brain analyses [2,4,6]. Once neural activity is reconstructed in a source space, functional connectivity can be estimated to characterize statistical dependencies among brain regions, and effective connectivity can be estimated to infer directed interactions within distributed neural networks [7,8,9]. In addition, graph-theoretical approaches provide a compact representation of brain network organization by quantifying measures, such as the clustering coefficient, path length, modularity, and centrality, that enable the study of large-scale brain topology in the contexts of both health and disease [10,11].

The reliability of EEG-derived connectivity and network measures is strongly influenced by preprocessing strategies, source modeling choices, and connectivity estimators [7,12]. Previous research has demonstrated that source localization results are generally more stable than downstream functional or effective connectivity estimates, highlighting the importance of rigorous methodological validation, which is necessary across forward models, inverse methods, software implementations, and experimental datasets [2,12]. This issue is particularly relevant because sensor-space connectivity is susceptible to confounding effects such as volume conduction and reference choice, whereas source-space analyses provide a more spatially interpretable basis for making network inferences [7,8,9].

Beyond conventional modeling approaches, machine learning and deep learning methods are increasingly being applied to EEG source imaging and brain network analyses for tasks such as classification, decoding, and automated feature extraction [13,14]. These data-driven approaches offer promising opportunities for improving analysis performance and clinical translation; however, they also introduce challenges related to interpretability, robustness, and generalizability across heterogeneous datasets [13,14]. Recent advances in self-supervised learning, transformer-based architectures, and large-scale representation learning further suggest that models pretrained on diverse EEG datasets may support transferable representations for downstream decoding tasks. Although these approaches have thus far been developed predominantly for sensor-level EEG and brain–computer interface applications, they provide a potentially important direction for source-space and network-level analyses, where anatomically informed source features may be combined with learned temporal and spectral representations [15,16,17,18].

Existing reviews have made important contributions by focusing on individual components of the workflow: practical EEG source imaging [2], source connectivity in magnetoencephalography (MEG)/EEG [9], functional-connectivity methods and interpretational pitfalls [8], graph-theoretical measures [10,11], and deep learning approaches for EEG [13,19]. Thus, these reviews primarily address source reconstruction, connectivity inference, network characterization, or data-driven modeling as focused methodological topics. The present review is complementary to these accounts because it evaluates how decisions and uncertainty at one stage propagate to, and constrain interpretation at, subsequent stages. Its distinctive contribution is to examine how decisions at each stage—from preprocessing and head modeling to inverse reconstruction, leakage mitigation, connectivity estimation, graph construction, and machine learning validation—constrain the interpretability of downstream results. Rather than proposing a new analytical method, the review provides a decision-oriented synthesis of cross-stage dependencies, error propagation, methodological trade-offs, and minimum validation requirements for source-level EEG and brain-network studies.

Therefore, this review synthesizes the methodological foundations and recent advances in EEG source localization and brain network analysis, with particular attention to functional and effective connectivity, graph-theoretical characterization, machine learning, deep learning, and clinical translation. In addition to describing established approaches, we provide practical guidance regarding method selection, identify unresolved sources of variability and disagreement, and propose priorities for robust and clinically translatable source-level EEG research.

## 2. The EEG Forward and Inverse Problems

An EEG source analysis is founded on the biophysical relationship between neuronal current generators within the brain and the electrical potentials recorded at the scalp. This relationship is mathematically described with the forward problem, which predicts scalp EEG signals from a known distribution of neural sources [1,2,3,4,5,6]. The forward model is commonly expressed as follows:

**V** = **LJ** + **ε**,

where **V** represents the measured scalp potentials, **J** denotes the unknown cortical current sources, **L** is the leadfield (gain) matrix describing the propagation of electrical activity through the head volume conductor, and **ε** represents measurement noise and modeling error [2,3,4,5,6,20]. More specifically, **V** ∈ ℝ^*M*×*T*^ denotes scalp potentials recorded at *M* electrodes over *T* time points; **L** ∈ ℝ^*M*×*P*^ is the leadfield matrix; **J** ∈ ℝ^*P*×*T*^ is the unknown source-current matrix; and **ε** ∈ ℝ^*M*×*T*^ denotes measurement noise and modeling error. Here, *P* represents the number of source parameters: *P* = *N* when dipole orientations are constrained to the cortical surface normal and approximately *P* = 3*N* when unconstrained three-dimensional dipole orientations are estimated for *N* candidate source locations. The leadfield matrix incorporates information about head geometry, tissue conductivity, and electrode locations, thereby serving as the fundamental link between neuronal activity and scalp recordings [2,4,5,20].

The accuracy of the forward model critically depends on the realism of the head volume conductor model. Modern forward modeling typically incorporates multiple tissue compartments—including the scalp, skull, cerebrospinal fluid, gray matter, and white matter—using subject-specific anatomical magnetic resonance imaging (MRI) [2,5,20,21]. Numerical approaches such as the boundary element and finite element methods, are widely used to account for the complex geometric and conductivity properties of the head [20,21,22]. Inaccurate tissue segmentation, conductivity assumptions, or electrode co-registration can introduce systematic localization errors, which emphasizes the importance of accurate anatomical modeling in source analyses [20,21,22,23].

From a practical perspective, head-model selection should be aligned with the study objective, available anatomical information, electrode density, and required localization accuracy. Spherical models are computationally efficient and can be adequate for teaching, simulation, or preliminary exploratory analyses, but their simplified geometry and conductivity assumptions limit anatomical realism. Boundary element method (BEM) models provide a widely used compromise between computational efficiency and anatomical fidelity, particularly when subject-specific MRI is available and the primary interest is cortical source imaging. Finite element method (FEM) models can incorporate complex tissue geometry and conductivity heterogeneity, including cerebrospinal fluid, anisotropic white matter, skull defects, lesions, or postoperative anatomy; however, they require more extensive segmentation, meshing, conductivity specification, computational resources, and quality control. Accordingly, BEM is often appropriate for conventional cortical source analyses, whereas FEM is particularly valuable when anatomically atypical structures or high-precision clinical localization are central to the research question [2,5,20,21,22,23].

In contrast, the inverse problem seeks to estimate the underlying cortical sources from the measured scalp potentials. Unlike the forward problem, which has a unique solution for a given head model, the inverse problem is fundamentally ill-posed because infinitely many source configurations can generate nearly identical scalp potential distributions [3,4,5]. Furthermore, the number of unknown source parameters greatly exceeds the number of EEG electrodes, and measurement noise and modeling uncertainties further increase estimation ambiguity [2,3,4,5]. Consequently, no unique inverse solution can exist without introducing additional assumptions or constraints [3,4,5].

To address the above ill-posedness, inverse modeling introduces regularization and prior information that restrict the solution space [3,4,5,24]. A standard Tikhonov *L*_2_-regularized inverse solution can be expressed as follows:

$$\hat{J}=\underset{J}{argmin}\left\{{\left\|V-LJ\right\|}_{F}^{2}+\frac{\lambda }{2}{\left\|WJ\right\|}_{F}^{2}\right\},$$

where ${\left\|\cdot \right\|}_{F}$ denotes the Frobenius norm, *λ* is the regularization parameter that balances data fit against solution complexity, and **W** is a weighting matrix that can encode depth weighting, spatial smoothness, or anatomical priors. The first term minimizes the discrepancy between the observed and model-predicted scalp potentials, whereas the second penalizes implausibly large or poorly conditioned source estimates. Different inverse algorithms can therefore be understood as imposing different regularization structures and priors, including minimum norm, noise normalization, spatial smoothness, sparsity, or probabilistic constraints. Rather than identifying a single mathematically correct solution, inverse methods estimate the most physiologically plausible source configuration that best explains the recorded EEG signals under predefined assumptions [2,3,4,5]. Consequently, source estimates inevitably depend on both the forward model and the selected regularization strategy [2,4,24].

Historically, EEG source localization was first addressed using equivalent current dipole (ECD) models, which assume that a small number of focal dipolar sources account for the observed scalp potentials [25,26]. Although ECD approaches remain useful for highly focal neural generators, they become less appropriate when multiple distributed or overlapping cortical sources contribute simultaneously to a recorded EEG [2,4,25], a limitation motivated the development of distributed source imaging techniques that estimate neural activity across the entire cortical surface, rather than assuming a small number of discrete generators [2,3,4,5]. The mathematical formulations, assumptions, and practical characteristics of these reconstruction algorithms are discussed in the following section.

Because every subsequent stage of a source-level EEG analysis relies on reconstructed cortical activity, uncertainties introduced during forward and inverse modeling inevitably propagate to all downstream analyses. Source localization errors can influence estimates of functional and effective connectivity, alter graph-theoretical network measures, and ultimately affect machine learning models developed for biomarker discovery or clinical prediction [7,8,9,10,11,12,13,14]. Recent comparative studies have demonstrated that differences in head modeling, inverse algorithms, software implementations, and parameter selection can produce substantial variability in source estimates and downstream connectivity measures, highlighting the need for rigorous validation using simulations, phantom experiments, multimodal imaging, and test–retest analyses [2,12,27].

From a computational neuroscience perspective, the forward and inverse problems therefore represent more than preliminary computational procedures, as they establish the quantitative framework that transforms scalp EEG data into anatomically interpretable cortical activity, providing the foundation for the source reconstruction, brain connectivity analyses, network modeling, and data-driven computational approaches discussed in this review [2,6].

## 3. EEG Source Reconstruction Algorithms

EEG source reconstruction algorithms build directly upon the forward and inverse formulations outlined above, with the goal of using a specified forward model to recover time-varying patterns of cortical activity from scalp-recorded data [2,3,4,5,6,26]. Broadly, these approaches can be classified as ECD models or distributed source imaging methods [2,4,24]. Because the EEG inverse problem is inherently ill-posed, no single reconstruction algorithm is universally optimal [3,4,5,24]. Instead, each method relies on distinct mathematical assumptions, physiological priors, and regularization strategies that produce characteristic trade-offs among localization accuracy, spatial resolution, robustness, and computational complexity (Table 1) [2,3,4,5,24]. Accordingly, method selection should be guided by the expected spatial configuration of neural generators, the signal-to-noise ratio and channel density of the EEG data, the required temporal or frequency-domain representation, and the intended downstream analysis.

### 3.1. Equivalent Current Dipole Models

ECD models assume that the observed scalp potentials are generated by a small number of focal dipolar sources whose locations, orientations, and amplitudes are optimized to best explain the recorded EEG signals [2,4,25,26]. Historically, ECD modeling has been widely used to localize relatively focal generators, such as epileptogenic zones or evoked responses, and it can provide intuitive and easily interpretable solutions when the number of active sources is relatively small and well separated [2,4,25].

However, real brain activity often involves many spatially distributed and simultaneously active sources, making ECD solutions highly sensitive to the assumed number of dipoles, initial parameter guesses, and optimization procedures [2,4,25]; moreover, ECD models are not well suited for characterizing large-scale network dynamics, in which activity is spread across extended cortical territories [2,4]. To address some of these limitations, various statistical and optimization techniques have been proposed to fit multiple dipole models [4,25]; nevertheless, distributed source imaging has emerged as the preferred approach for investigating large-scale cortical activity and brain networks [2,4,24].

### 3.2. Minimum-Norm and Low-Resolution Electromagnetic Tomography (LORETA)-Based Distributed Source Imaging

Distributed source imaging methods define a large set of candidate source locations across the cortical surface (or volume) and estimate activity at all locations simultaneously [2,3,4,5,24]. A central example is minimum-norm estimation (MNE), which identifies the source distribution with the minimum overall power that best explains the observed scalp potentials under the constraints of the forward model [3,20]. Minimum-norm solutions can be computed efficiently using the leadfield matrix and noise covariance, making them computationally tractable, and they are widely implemented in clinical and research toolboxes [3,28,29]. However, because MNE minimizes overall source power, it exhibits a depth bias that tends to favor superficial cortical sources unless depth-weighting or normalization procedures are applied [2,3,24].

LORETA-family algorithms extend MNE by introducing spatial smoothness constraints that favor neighboring cortical sources with similar current densities [20,30,31]. Consequently, these methods produce spatially continuous activation patterns that are often more physiologically plausible explanations of distributed cortical activity than those produced with other MNE algorithms, but that plausibility comes at the expense of reduced spatial resolution [2,20,30]. Excessive smoothness can merge adjacent generators, increase source leakage, and consequently bias downstream connectivity analyses [8,9,12].

Dynamic statistical parametric mapping extends the classical MNE framework by normalizing the source estimates with respect to the estimated noise covariance, thereby improving statistical interpretability and partially compensating for the superficial source bias inherent in conventional MNE [32]. Within the LORETA family, the standardized LORETA (sLORETA) theoretically achieves zero localization error for a single source in ideal noise-free conditions, whereas the exact LORETA (eLORETA) reduces localization bias by imposing an exact localization constraint while preserving a distributed estimation framework [30,31]. Recently, sparse and hybrid regularization strategies have been developed to balance spatial smoothness with focal source localization, thereby improving spatial specificity while maintaining numerical stability [27,33].

### 3.3. Beamforming and Spatial Filter Approaches

Beamforming algorithms construct linear spatial filters that selectively pass activity from a target source location while suppressing signals originating from other brain regions [5,26,34]. Representative beamforming approaches include linearly constrained minimum variance (LCMV), synthetic aperture magnetometry (SAM), and dynamic imaging of coherent sources (DICS), all of which estimate spatial filter weights using the leadfield matrix and sensor covariance information [34,35,36]. Although LCMV and SAM are primarily applied to time-domain source reconstruction, DICS operates in the frequency domain and is particularly well suited for investigating oscillatory brain activity and frequency-specific functional connectivity [34,35,36]. Beamforming methods can provide high spatial resolution for selected time windows or frequency bands while effectively attenuating interference from non-target sources [5,34,35,36].

On the other hand, beamformer performance critically depends on accurate covariance estimation, dimensionality reduction strategies, and the signal-to-noise ratio of the data; inaccurate or rank-deficient covariance matrices and strong collinearity among channels can destabilize the spatial filters and compromise localization accuracy [5,34]. A particular limitation of spatial-filter beamformers arises when multiple neural generators are strongly correlated or phase locked. LCMV beamformers estimate filter weights from the sensor covariance matrix while imposing a unit-gain constraint at a target location. When two spatially distinct sources exhibit highly correlated activity, their scalp projections become difficult to distinguish in the covariance structure. The variance-minimization step can then treat activity from one correlated source as interference when constructing the filter for the other source, leading to attenuation or partial cancellation of one or both source estimates, which can produce false-negative activation maps, distort source amplitude estimates, and bias downstream connectivity analyses. Mitigation strategies include careful covariance-window selection, appropriate regularization, reduced source-space dimensionality, dual-source or vector beamforming approaches, and sensitivity analyses using simulated correlated sources [5,34,35,36]. Consequently, beamforming approaches are frequently used as a complement to minimum-norm and LORETA-based distributed source imaging in task-related activation mapping and oscillatory network analyses [2,5,34,35,36]. Because covariance estimation, source correlation, and spatial-filter design can alter the reconstructed time series, these choices should be considered when beamformer outputs are used for downstream connectivity or network analyses.

### 3.4. Bayesian and Sparse Inference Frameworks

Bayesian source reconstruction imposes explicit prior distributions on the source activity and computes posterior distributions given the observed data, thereby providing a probabilistic formulation of the EEG inverse problem [5,27,33]. Unlike deterministic inverse methods, Bayesian frameworks naturally provide posterior uncertainty estimates, facilitating probabilistic interpretation and integration with multimodal neuroimaging data [5,23]. A representative example is the multiple sparse priors framework, which partitions the source space into multiple candidate regions and assumes sparse activation patterns within them, estimating source distributions by minimizing negative variational free energy within a Bayesian model selection framework [27,33]. Such Bayesian and sparsity-promoting approaches are particularly well suited for capturing multifocal but localized activation patterns, and they can improve spatial resolution relative to conventional smooth minimum-norm solutions [27,33].

At the same time, Bayesian and sparse approaches are highly dependent on the choice of priors, hyperparameter tuning, and optimization procedures, and they are often computationally demanding [27,33]. Consequently, these methods are primarily adopted in advanced source imaging and high-resolution network analyses. Standardized algorithms such as MNE, LORETA-family methods, and beamforming remain dominant in routine clinical practice and many conventional EEG research applications [2,28,29,30,31,32,33,34,35,36].

### 3.5. Practical Considerations and Consistency Across Algorithms

The selection of an EEG source reconstruction algorithm should be guided by the scientific objective, expected spatial distribution of neural generators, data quality, and requirements of any downstream analyses, because each inverse method carries distinct methodological trade-offs [2,4,5,6,24]. Comparative studies have demonstrated that different combinations of forward models, inverse algorithms, regularization strategies, and software implementations can produce substantially different source localization and connectivity estimates, even when they are applied to the same EEG datasets [12,27]. Notably, Mahjoory et al. showed that EEG source localization is generally more consistent than source-level functional connectivity estimates across different analysis pipelines, suggesting that methodological variability accumulates throughout the processing workflow, rather than arising solely from the inverse solution itself [12]; these findings emphasize that algorithm selection should always be considered within the context of the complete source imaging pipeline.

In practice, several methodological factors require careful consideration. The choice between ECD and distributed source models should be determined by the anticipated source configuration and the underlying research or clinical question [24,25]; likewise, the regularization strategy, including the balance between spatial smoothness and sparsity and the incorporation of anatomical or probabilistic priors, directly influences localization accuracy, spatial resolution, and source leakage [24,27,30,31,33]. Data characteristics, such as the number of EEG channels, signal-to-noise ratio, recording duration, and whether the analysis targets resting-state or task-related activity, further affect the performance of covariance estimation and inverse reconstruction [5,15,26,34]. The availability of subject-specific anatomical MRI, as opposed to template head models, can further improve localization accuracy by enabling more realistic forward modeling [20,21,22,23]. Moreover, differences in default parameters, numerical implementations, and preprocessing pipelines among commonly used software platforms—including EEGLAB, MNE-Python, Brainstorm, and FieldTrip—can introduce additional variability into source estimates, underscoring the importance of transparent reporting and reproducible analysis workflows [12,28,29].

Ultimately, EEG source reconstruction algorithms should not be regarded as independent computational tools but rather as integral components of a unified computational framework that encompasses forward modeling, inverse estimation, preprocessing, connectivity analyses, graph-theoretical modeling, and machine learning [2,5,6,7,10,11,12,13,14]. Because reconstructed source time series constitute the input to connectivity analyses, algorithm selection should be evaluated not only in terms of localization performance but also in terms of its implications for source leakage, temporal fidelity, and the validity of downstream connectivity and network measures [8,9,12]. Careful algorithm selection, rigorous validation, and transparent reporting are therefore essential for obtaining reliable, reproducible, and biologically meaningful data from source-level EEG analyses [2,12,27]. Accurate and stable source reconstruction provides the foundation for the reliable estimation of functional and effective connectivity, which in turn enables meaningful characterization of large-scale brain network organization [7,8,9,10,11,12]. The following section examines how reconstructed source activity can be used to infer interactions among distributed brain regions through functional and effective connectivity analyses.

## 4. From Source Activity to Functional and Effective Connectivity

Once neural activity has been reconstructed in source space, the next methodological stage is to characterize interactions among cortical regions through functional and effective connectivity analyses [7,8,9,37]. Functional connectivity describes statistical dependencies between neural signals, typically quantified using measures of correlation, coherence, amplitude coupling, or phase synchronization, whereas effective connectivity aims to infer directed interactions within distributed neural networks [7,8]. Because these measures are calculated from reconstructed source time series rather than directly from sensor recordings, their validity and interpretability depend jointly on source-reconstruction quality, residual source leakage, the selected connectivity estimator, and subsequent network-construction choices (Table 2) [8,9,12]. Accordingly, connectivity measures should be selected according to the scientific question, the expected temporal and spectral characteristics of the interaction, the need to estimate directionality, and the susceptibility of the data to leakage-related confounding. In practice, functional connectivity is generally appropriate when the objective is to characterize undirected statistical coupling or large-scale synchronization, whereas effective connectivity approaches are preferable when a specific hypothesis concerns directed interactions and their underlying dynamical or causal mechanisms.

### 4.1. Functional Connectivity in a Source Space

Functional connectivity in source space is commonly quantified using measures of linear or nonlinear statistical dependence between reconstructed source time series, analyzed either directly in the time domain or in the frequency domain following spectral decomposition [7,8,9,37,38]. Classical time-domain approaches include Pearson correlation and cross-correlation, whereas frequency-domain measures such as magnitude-squared coherence, phase-locking value, and related phase-based indices characterize oscillatory coupling within specific frequency bands [8,9,38]. A typical source-space connectivity analysis pipeline involves extracting reconstructed source time series, computing time- or frequency-domain representations depending on the selected connectivity measure, estimating pairwise connectivity, and performing statistical analyses at the group level [8,9].

A principal motivation for estimating connectivity in source space is to reduce the influence of volume conduction and common-reference effects, both of which can generate spurious zero-lag correlations in sensor-space EEG data [8,9,39]. Nevertheless, source reconstruction does not completely eliminate signal leakage, particularly when spatially smooth inverse solutions, low-density EEG recordings, or highly correlated cortical sources are present [9,12]. Measures designed to reduce sensitivity to instantaneous mixing include the phase-lag index (PLI), weighted PLI (wPLI), and the imaginary part of coherency, all of which discount zero-phase interactions [39,40,41]. Unlike conventional coherence, imaginary coherency retains only the imaginary component of the cross-spectrum and thereby suppresses instantaneous, zero-lag coupling that is commonly produced through volume conduction or residual source leakage [39,40,41]. However, these approaches can underestimate physiologically meaningful near-zero-lag interactions and may have reduced sensitivity under low signal-to-noise conditions [8,39,40,41]. Alternative approaches, including amplitude-envelope correlation with orthogonalization or other leakage-correction procedures, provide complementary strategies but introduce their own assumptions and potential signal distortions [42,43]; therefore, no single connectivity estimator is universally optimal; analyses should justify the selected estimator and, where feasible, evaluate whether key findings are robust across plausible leakage-mitigation strategies.

### 4.2. Effective Connectivity and Directed Interactions

Effective connectivity aims to characterize directed interactions among brain regions within a framework of dynamic systems or causal inferences [7,44]. For EEG source data, multivariate autoregressive (MVAR) models provide the basis for widely used measures such as Granger causality, directed transfer function, and partially directed coherence, which estimate frequency-specific directional interactions under assumptions of linearity and stationarity [45,46,47]; these approaches have been successfully applied in many cognitive and clinical paradigms, but their performance critically depends on appropriate model-order selection, the stationarity of the analyzed signals, and the dimensionality of the reconstructed source space [8,45,46,47].

Residual source leakage is also a critical concern for MVAR and Granger-causality analyses. Leakage can mix activity from one reconstructed source into another, creating a shared temporal structure that violates the assumption that each input time series represents a distinct neural generator. In an MVAR model, this mixing can produce apparent lagged dependencies and spurious directed edges, particularly when model order, signal-to-noise ratio, source correlation, or dimensionality are not adequately controlled. Source-space Granger-causality results should therefore be interpreted cautiously and evaluated using leakage-aware reconstruction procedures, appropriate model-order selection, surrogate-data testing, and sensitivity analyses across plausible source-reconstruction and connectivity pipelines [8,9,12,45,46,47].

Beyond MVAR-based approaches, model-driven frameworks such as dynamic causal modeling (DCM) estimate effective connectivity by fitting biophysically informed generative models to source-level EEG data within a Bayesian inference framework [7,44]. DCM uses biologically informed generative models of interacting neuronal populations to enable the hypothesis-driven inference of directed connectivity and task-dependent network modulation [44]. However, DCM is computationally demanding, typically limited to relatively small networks, and highly sensitive to model specification and prior assumptions, restricting its routine application to large-scale EEG network analyses [7,44]. Consequently, effective connectivity analyses should be supported by simulation studies, multimodal validation, and comparison with structural connectivity whenever possible [8,9,12]. Because directed estimates are especially sensitive to source leakage, signal-to-noise ratio, model order, and violations of stationarity, their interpretation should be supported by sensitivity analyses and, where possible, convergent evidence from alternative models or modalities [8,9,12,44,45,46,47].

### 4.3. Sensor- Versus Source-Space Connectivity

Numerous simulation and empirical studies have demonstrated important differences between connectivity estimated in a sensor space and that reconstructed in a source space [8,9,12,38]. Sensor-space connectivity is computationally straightforward and does not require head modeling or source reconstruction; however, it is strongly affected by volume conduction, reference selection, and common-source artifacts [8,9,39,40,41]. In contrast, source-space connectivity provides improved anatomical interpretability and enables connectivity estimates to be mapped onto cortical regions or anatomical parcellations, but its accuracy depends on the quality of the forward model, inverse solution, and degree of spatial leakage introduced during source reconstruction [2,9,12].

Although source-space analyses substantially reduce spurious zero-lag interactions, leakage-induced dependencies are not completely eliminated, particularly when smooth distributed inverse methods or low-density EEG montages are analyzed [9,12,39,40,41,42,43]; furthermore, preprocessing strategies, artifact rejection procedures, frequency-band selection, and source parcellation schemes can influence sensor- and source-space analyses differently, making direct comparisons between them challenging [8,9,12]. Accordingly, source-space connectivity should not simply be regarded as a corrected version of sensor-space connectivity, but rather as a complementary representation with distinct assumptions, advantages, and limitations [8,9,12].

### 4.4. Reliability, Validation, and Methodological Considerations

The reliability of source-space functional and effective connectivity estimates depends on multiple methodological factors, including preprocessing pipelines, source reconstruction algorithms, connectivity estimators, and network construction procedures [8,9,12]. Test–retest studies and multiple pipeline comparisons have consistently demonstrated that connectivity estimates are generally less reproducible than source localization itself, with reliability varying according to the frequency band, connectivity metric, and network topology [12]. Phase-based measures that minimize volume conduction effects can exhibit low reproducibility because of their sensitivity to noise and signal non-stationarity; amplitude-based measures are often more stable than phase-based measures, but they remain susceptible to common-input effects [39,40,41,42,43].

Reliable connectivity estimation therefore requires validation using complementary approaches, including simulations with known ground truth, surrogate-data testing, test–retest assessment, cross-validation across independent datasets or recording sessions, and comparison with structural connectivity or other neuroimaging modalities such as functional MRI (fMRI) and MEG [8,9,12,37,38]. Connectivity validation should assess not only the performance of the selected estimator but also the effects of upstream preprocessing, forward-modeling, inverse-reconstruction, and source-parcellation choices. In addition, network-construction decisions, including thresholding, weighting, and correction for multiple comparisons, can substantially influence the resulting connectivity matrix and should be reported transparently [8,9,12].

Ultimately, source-space connectivity provides the essential link between reconstructed neural activity and large-scale brain-network analyses [37,38]. Connectivity matrices derived from source-space EEG can be represented as graphs, enabling quantitative characterization of network topology, integration, segregation, and hub organization. However, graph-theoretical measures depend on the properties of the input connectivity matrix, including the selected connectivity estimator, source parcellation, leakage-correction strategy, and network-construction or thresholding procedure [8,9,12,37,38]. Therefore, graph-theoretical findings should be interpreted as outputs of the complete analytical pipeline, rather than as direct and method-independent representations of brain-network organization. The following section reviews graph-theoretical methods that transform connectivity matrices into brain networks and quantify their topological organization [37,38].

## 5. Graph-Theoretical Analysis of Source-Level Brain Networks

Graph theory provides a compact and mathematically rigorous framework for representing and quantifying large-scale brain networks derived from source-space EEG connectivity [10,11,37,38,48,49]. In this framework, cortical regions or source parcels are modeled as nodes, and functional or effective connectivity estimates between them constitute edges, yielding a graph on which a range of topological metrics can be computed [10,11,48]. Applying a graph-theoretical analysis to source-level EEG networks enables the characterization of key properties such as integration, segregation, hub organization, modularity, and robustness, thereby linking neural dynamics to cognitive function and clinical status [10,11,48,49].

### 5.1. Constructing Brain Graphs from Source-Space Connectivity

The construction of brain graphs from source-space EEG data typically proceeds by parcellating the cortical surface into a set of regions of interest (ROIs), extracting regional source time series, estimating functional or effective connectivity between ROI pairs, and representing the resulting connectivity matrix as a graph [10,11,48,49]. Nodes represent cortical regions or source parcels, and edges represent estimated interactions. Graphs may be weighted, in which edge weights retain the magnitude of connectivity estimates; or binary, in which edges are retained or removed according to a thresholding rule. Weighted graphs preserve more information but remain sensitive to differences in connectivity scale, whereas binary graphs facilitate comparisons across studies but can be highly sensitive to threshold selection [10,48,49].

Thresholding should be selected according to the scientific question and the properties of the connectivity measure. Absolute thresholding retains edges above a fixed connectivity value and can preserve potentially meaningful differences in overall connection strength; however, it produces networks with different densities across participants or groups, which can confound graph-metric comparisons. Proportional thresholding retains a fixed proportion of the strongest connections and facilitates comparison of networks with matched density, but it may remove biologically relevant weak connections or retain weak and potentially spurious edges when overall connectivity is low. Consequently, neither approach is universally preferable. When feasible, analyses should report weighted results and evaluate whether conclusions remain stable across a justified range of proportional or absolute thresholds, with an appropriate null-model framework [10,11,48,49].

### 5.2. Measures of Segregation and Integration

Graph-theoretical analyses of brain networks are often organized around the concepts of segregation and integration [10,11,48]. Segregation refers to the tendency of nodes to form clusters or modules that support specialized information processing, and integration reflects the capacity of a network to combine information across distributed regions [10,11,48]. Common measures of segregation include the clustering coefficient, which quantifies the prevalence of triangles or local neighborhood connectivity, and modularity, which assesses the strength of a community structure relative to a random reference network [10,11,50].

Measures of integration include the characteristic path length, global efficiency, and related metrics that capture the ease of communication across the entire network [10,11,48]. Many EEG-derived functional networks have been shown to exhibit small-world properties, characterized by high clustering combined with short path lengths relative to random graphs, a topology thought to support efficient information processing at a low wiring cost [51,52]. Deviations from small-world organization—such as increased path length or decreased clustering—have been reported in various neurological and psychiatric conditions, suggesting that alterations in the integration–segregation balance might serve as network-level biomarkers [10,11,48,51].

Among commonly reported graph measures, global efficiency, characteristic path length, clustering coefficient, and modularity are useful for characterizing integration and segregation, but their reliability varies with data quality, network density, parcellation, connectivity estimation, and thresholding procedures. For this reason, isolated changes in a single metric should not be interpreted as definitive evidence of altered network organization without convergent changes across related metrics, robustness analyses, and comparison with appropriate null models [10,11,12,48,49].

### 5.3. Centrality, Hubs, and Rich-Club Organization

Graph metrics of centrality aim to identify nodes that are structurally or functionally important for network communication [10,11,48,49]. Common centrality measures include degree (number of connections per node), betweenness (fraction of shortest paths passing through a node), and the eigenvector or page rank, which capture influence in relation to the importance of neighboring nodes [10,11,48]; nodes with high centrality are often interpreted as hubs that could play pivotal roles in integrating information across modules and maintaining network resilience [10,11,48,49].

At a higher level of organization, the rich-club phenomenon describes the tendency of high-degree or high-centrality nodes to be more densely interconnected with each other than expected by chance, forming a “core” subnetwork that supports global integration [53]. Source-level EEG studies have reported hub and rich-club structures in resting-state and task-related networks, with hub regions frequently overlapping association cortices involved in high-level cognitive functions [11,48,53]. Disease-related changes in hubness and rich-club connectivity might reflect the selective vulnerability of highly connected regions and provide insight into how local pathology propagates across the network [10,11,48,53].

Centrality and rich-club measures can offer clinically meaningful information about potential hub vulnerability, but they are particularly sensitive to node definition, edge density, and the treatment of weak connections; therefore, hub-related findings should be replicated across plausible graph-construction choices before being interpreted as stable neurobiological markers [10,11,48,49].

### 5.4. Network Dynamics, Robustness, and Clinical Applications

Graph-theoretical analyses of source-level EEG networks are not limited to static connectivity; dynamic approaches examine how network topology changes over time or across cognitive states [10,11,48,49]. Time-resolved graph analyses involve computing connectivity and graph metrics within sliding windows or event-related epochs, enabling the characterization of transient changes in integration, segregation, and hub structure during tasks or transitions between vigilance states [10,11,48]. Such dynamic network analyses can reveal state-dependent reconfiguration patterns that could be obscured in static, time-averaged networks [10,48].

The robustness and resilience of brain networks can be assessed by simulating node or edge removal and examining the effects on global efficiency, path length, or modular structure analyses [10,11,48], which address questions about the ways in which brain networks respond to focal lesions, degenerative processes, or targeted interventions, and they can guide hypotheses about compensatory reorganization and the network-level mechanisms of recovery [48,49]. Clinically, graph-theoretical metrics derived from source-level EEG data have been associated with cognitive performance, disease severity, and treatment response in conditions such as epilepsy, dementia, traumatic brain injury, and mood disorders, though the findings remain heterogeneous and methodologically sensitive [10,11,48].

The available literature does not justify a universal ranking of graph metrics across disorders. Global efficiency, characteristic path length, clustering coefficient, and modularity are useful because they have relatively direct network interpretations and are widely reported; however, their direction and magnitude remain dependent on connectivity estimation, graph density, parcellation, and null-model selection. Centrality and rich-club measures can generate biologically informative hub hypotheses, but they are particularly sensitive to node definition and weak-edge treatment and should not be interpreted as stable clinical markers without stability analyses and independent replication. Table 3 summarizes these metric-specific trade-offs, the current level of evidence, and the corresponding reporting and validation practices.

Graph-theoretical findings should therefore be interpreted as outputs of the complete analytical pipeline rather than as direct and method-independent representations of brain-network organization. Clinically meaningful inference requires sensitivity analyses across plausible source-reconstruction, connectivity-estimation, parcellation, and thresholding choices, together with appropriate null models, repeated-recording reliability where feasible, and independent validation. Transparent reporting of these analytical decisions is essential before graph metrics can be considered reliable biomarkers or used to support clinical inferences. The following section examines how source-level features, connectivity matrices, and graph-derived measures can be incorporated into machine learning and deep learning models.

## 6. Machine Learning and Deep Learning for EEG Source and Network Analyses

Machine learning and deep learning have become increasingly important in EEG research because they provide flexible frameworks for extracting patterns from high-dimensional, noisy, and nonstationary data [13,19,54]. In the context of source-level EEG data, these approaches have been applied to classification, prediction, and biomarker discovery and are increasingly being explored for source reconstruction and brain-network analyses [13,14,19]. Their appeal lies in their ability to integrate temporal, spectral, spatial, connectivity, and graph-derived features within unified predictive frameworks [13,14]. Recent developments include self-supervised learning, foundation-model approaches, transformer-based architectures, and large-scale representation learning; these methods aim to learn transferable EEG representations from large-scale unlabeled and heterogeneous datasets and may reduce dependence on manually engineered features; however, their utility for source-level EEG and clinically heterogeneous populations requires rigorous external validation and careful assessment of domain shift [13,14,19,54,55,56].

Recent EEG representation-learning studies have explored attention-based convolutional networks, adaptive tokenization and embedding strategies, large-scale pretrained brain models, and pretrained transformer models for learning transferable representations across tasks and datasets. For example, BCINetV1 integrates temporal and spectral attention for motor-imagery EEG decoding, adaptive tokenization frameworks aim to improve interpretability through structured EEG embeddings, large brain models have been proposed to learn generic representations from extensive brain–computer interface (BCI) datasets, and EEGPT applies pretrained transformer architectures to support universal EEG representation learning [15,16,17,18]. The above advances reflect a shift from task-specific feature engineering toward pretrained and transferable representations.

These frameworks serve different purposes within EEG representation learning and should not be regarded as interchangeable. Attention-based convolutional models, such as BCINetV1, primarily refine task-specific temporal and spectral decoding. Adaptive tokenization and embedding methods focus on constructing interpretable input representations, whereas large pretrained brain models and transformer-based approaches aim to learn transferable representations from extensive and heterogeneous EEG datasets. Their relevance to source-level EEG remains indirect because most evidence is derived from sensor-level EEG or BCI datasets. Future studies should determine whether these representations remain robust to differences in head modeling, inverse solutions, source parcellation, acquisition systems, and patient populations, and whether they outperform well-validated feature-based baselines under participant-level partitioning and independent external validation. Until such evidence is available, their clinical utility for source-level EEG and brain-network applications should be considered provisional. Representative machine learning and deep learning approaches, their typical input representations, strengths, limitations, and applications in source-level EEG and brain-network analyses are summarized in Table 4.

In conventional machine learning pipelines, source-level features are extracted from reconstructed cortical activity and then used for supervised or unsupervised analyses [13,14]. Common input features include source power, band-limited amplitude, phase-based coupling indices, connectivity matrices, graph-theoretical metrics, and spatio-temporal summaries of regional activity [10,11,13,14]. Classical algorithms such as support vector machines, random forests, logistic regression, and gradient boosting re-main widely used because they are relatively interpretable, computationally efficient, and suitable for moderate-sized datasets [13,19]. However, their performance depends strongly on feature engineering, preprocessing quality, and the validity of the source-reconstruction pipeline [12,13].

Deep learning extends this framework by learning hierarchical representations directly from source-level time series, connectivity matrices, or graph-structured data [13,19,54]. Convolutional neural networks have been used to model spatial and temporal patterns in source-reconstructed signals, whereas recurrent architectures and transformer-based models are increasingly being explored for sequence modeling and long-range temporal dependencies [19,54]. In parallel, graph neural networks have emerged as a natural approach to EEG network analyses because they operate directly on connectivity graphs and can exploit topological structure in source-level brain networks [14,55]; these models are particularly attractive when the goal is to integrate anatomical, functional, and graph-based information within a unified predictive framework [14,55].

Graph neural networks are particularly vulnerable to overfitting in small clinical EEG datasets because model capacity can exceed the number of independent participants and because graph topology may encode site-, acquisition-, or pipeline-specific features. Mitigation strategies include participant-level cross-validation, weight decay, dropout, early stopping, graph or feature augmentation, edge dropout, node-feature masking, simplified architectures, and pretrained or self-supervised representations when sufficiently large external data are available. Such procedures should be combined with external validation rather than relying solely on random splits of epochs or segments from the same participants [13,14,19,54,55,56].

Despite their advantages, data-driven approaches to EEG analysis also introduce important methodological challenges [13,19]. Deep learning models are often data-hungry, sensitive to dataset imbalance, and vulnerable to overfitting when applied to small or heterogeneous EEG cohorts [13,19,54]. In source-level analyses, performance can also be affected by upstream errors in preprocessing, forward modeling, inverse reconstruction, connectivity estimation, and graph construction, meaning that machine learning models can inadvertently learn methodological artifacts rather than genuine neurobiological signals [12,13].

Common data-leakage scenarios include splitting epochs, windows, trials, or data segments from the same participant across training and test sets; fitting normalization, artifact-rejection, feature-selection, dimensionality-reduction, or hyperparameter-tuning procedures using information from the full dataset before data partitioning; and allowing repeated sessions, related recordings, or site-specific acquisition characteristics to appear in both training and test data. Such leakage can substantially inflate performance estimates without improving generalizability; therefore, robust evaluation should use participant-level or site-level data partitioning, fit all preprocessing and feature-selection procedures within training data only, use nested cross-validation for model selection, and preserve an independent external test set whenever feasible [13,19,56].

Robust machine learning evaluation should also include sensitivity analyses across plausible source-reconstruction, connectivity-estimation, and graph-construction pipelines, together with ablation testing, calibration assessment, class-imbalance handling, and transparent reporting of all analytical decisions. External validation across independent datasets or sites is essential before clinical utility or generalizability can be claimed [12,13,19,56].

Interpretability is another central issue [13,19,56]. Although deep learning can achieve high classification performance, its internal representations are often difficult to relate to specific neurophysiological mechanisms [19,54], a limitation that becomes especially relevant in clinical applications, where explainability and trustworthiness are critical for adoption [56]. Methods such as saliency mapping, occlusion analyses, attention mechanisms, and post hoc feature attribution have therefore been used to identify which source regions, time windows, frequencies, or graph nodes contribute most strongly to a model prediction [54,56]. In EEG source and network analyses, such approaches could help bridge the gap between predictive modeling and mechanistic interpretation [13,14,56].

From a translational perspective, machine learning and deep learning are being explored for seizure classification, epileptogenic-zone estimation, cognitive-state decoding, disease stratification, and biomarker development [13,19,54]. In epilepsy, source-level features and network metrics can improve characterization of abnormal dynamics and could complement conventional source localization in presurgical evaluations [2,12,13]. More broadly, these approaches could support personalized medicine by identifying subject-specific signatures that are difficult to capture with group-level statistics alone [13,19]. However, clinical translation will depend on reproducibility across cohorts, robustness to acquisition differences, and integration with established neurophysiological knowledge [12,13,19,56].

Overall, machine learning and deep learning offer powerful extensions to EEG source and network analyses, but their value depends on the quality of upstream source reconstruction, the appropriateness of feature design, and the rigor of validation [12,13,19]. Rather than replacing biophysical modeling, learning-based approaches should be regarded as complementary tools that build upon anatomically grounded source-imaging and network-neuroscience frameworks [2,13,19]. When integrated within a rigorously validated computational pipeline, these approaches can support prediction, biomarker discovery, and clinical decision support [13,14,19,56].

## 7. Clinical and Cognitive Neuroscience Applications

EEG source imaging and source-level network analyses have become increasingly important in clinical neurology [2,6,57,58]. By reconstructing epileptiform activity in a source space, EEG source imaging can improve the localization of epileptogenic zones and provide anatomically interpretable information that complements conventional scalp EEG and structural MRI [2,57,58]. When combined with graph-theoretical analyses, source-level connectivity measures further reveal widespread alterations in functional networks that extend beyond the seizure onset zone, underscoring how epilepsy is network-based [10,11,48,58]. The above findings support source-space EEG as a noninvasive and comparatively accessible adjunct for presurgical planning, longitudinal monitoring, and treatment assessment. Prospective surgical epilepsy studies indicate that EEG source imaging can provide clinically meaningful localization information when interpreted alongside structural imaging, scalp EEG, intracranial EEG, and other electrophysiological findings [57,58]. Among current applications, presurgical epilepsy evaluation remains one of the most mature clinical settings because source imaging can be compared with seizure semiology, scalp EEG, structural MRI, intracranial EEG, and postoperative outcomes. Even in this setting, source-space findings should be interpreted as complementary evidence within a multimodal clinical assessment rather than as stand-alone determinants of surgical decisions [2,57,58].

Beyond epilepsy, source-level EEG and network analyses are increasingly being investigated in dementia, traumatic brain injury, schizophrenia, depression, and other neuropsychiatric conditions [59,60,61]. In these settings, graph-theoretical measures such as clustering, path length, efficiency, and hubness have been associated with cognitive performance, symptom severity, and disease progression. However, findings remain heterogeneous because clinical phenotypes and disease mechanisms vary substantially across and within these conditions; medication effects, comorbidity, injury characteristics, and disease stage can alter EEG activity; and differences in recording conditions, preprocessing, source reconstruction, connectivity estimation, and graph construction can substantially affect derived network metrics [10,11,12].

The maturity of evidence differs substantially across clinical applications (Table 5). In presurgical focal epilepsy, the most defensible role of source imaging is as an adjunct that contributes to a convergent localization hypothesis alongside semiology, scalp EEG, structural imaging, and, when indicated, intracranial EEG. Clinically meaningful validation should include concordance with the presumed epileptogenic zone, resection site, and postoperative seizure outcome. In dementia, traumatic brain injury, schizophrenia, and depression, source-level network measures remain primarily research tools. Their near-term value is more likely to lie in longitudinal stratification, monitoring, or prediction of treatment response than in stand-alone diagnosis; these applications require prospective validation demonstrating incremental value beyond clinical, cognitive, and imaging assessments. Across applications, translation also requires transparent reporting of uncertainty and explicit, application-specific validation targets.

Source-level EEG analyses also offer important advantages for developmental and cognitive neuroscience because they enable high-temporal-resolution investigations of cortical dynamics in anatomically defined regions [2,6,62]. In developmental studies, source-space connectivity analyses have increasingly been used to characterize the maturation of functional brain networks in infants and children, revealing age-dependent changes in network integration and segregation that are associated with cognitive development [63]. In cognitive paradigms, source-level oscillatory activity and connectivity analyses support detailed mapping of task-related network reconfiguration, including transient changes in frontoparietal and sensory association networks during attention, memory, and emotion processing [9,37,62]. The above applications highlight the utility of source-space EEG as a bridge between classical event-related potentials and modern network neuroscience [2,62].

Across both clinical and cognitive-neuroscience applications, routine implementation remains limited by several technical and methodological barriers [2,8,9,12,62]. Differences in head modeling, inverse algorithms, connectivity estimators, parcellation schemes, graph construction procedures, electrode density, signal quality, artifact rejection, and recording conditions can substantially affect source-localization results, connectivity maps, and derived network metrics [12,20,21,22,23,48]. Clinical workflows must also address acquisition time, MRI availability, computational resources, software interoperability, quality assurance, operator expertise, and the need for transparent and reproducible reporting.

Reliable clinical translation therefore requires harmonized acquisition and preprocessing protocols, prespecified analysis pipelines, robustness analyses across plausible methodological choices, prospective validation in independent and clinically representative cohorts, and demonstration of incremental clinical utility. Source-level EEG findings should be interpreted alongside clinical history, scalp EEG, structural imaging, neuropsychological assessment, and other electrophysiological or neuroimaging data, rather than as stand-alone diagnostic or therapeutic decision tools.

Furthermore, integrating source-level EEG analyses with other modalities—such as MRI, MEG, and fMRI—offers promising avenues for multimodal clinical and cognitive neuroscience [32,62,64,65,66]. Anatomically informed source imaging can be integrated with structural MRI, diffusion MRI, MEG, fMRI, and metabolic imaging to yield a more comprehensive picture of brain organization in health and disease than any modality could produce alone [62,64,65,66]. In epilepsy, for instance, convergence between EEG source imaging, graph-theoretical network alterations, and structural lesions can strengthen hypotheses regarding epileptogenic networks and inform presurgical decision-making [57,58,60]. In cognitive neuroscience, multimodal approaches can help link fast electrophysiological dynamics to slower hemodynamic responses and structural constraints, thereby advancing mechanistic models of large-scale brain function [64,65]. Collectively, these applications illustrate the growing role of source-level EEG analyses and network neuroscience in translational research. Continued methodological standardization, validation across independent cohorts, and integration with multimodal neuroimaging will be essential for realizing their full clinical and scientific potential [2,12,62,64,65].

## 8. Current Challenges and Future Directions

Despite substantial methodological progress, EEG source localization and brain-network analyses still face interdependent challenges that limit their interpretability and translational impact. Model uncertainty at the levels of preprocessing, forward modeling, and inverse reconstruction remains a major concern because differences in head models, tissue conductivities, electrode montages, source-space definitions, inverse algorithms, and regularization strategies can yield different source estimates and downstream connectivity maps, even for identical datasets [2,3,4,5,6,12,20,21,22,23,24,25,26,27,28,29,30,31,32,33,34,35,36,67]. The above uncertainties propagate through the complete analytical workflow: reconstruction error and source leakage influence connectivity estimation, connectivity matrices determine graph-theoretical measures, and source-level or network-derived features can alter machine learning outputs and clinical inferences [8,9,12,39,40,41,42,43]; therefore, validation should assess the robustness of conclusions across plausible end-to-end pipelines rather than evaluating individual steps in isolation.

A second challenge lies in integrating biophysical modeling with data-driven approaches such as machine learning and deep learning. Complex models, including deep architectures and graph neural networks, can achieve strong performance for classification, decoding, and biomarker discovery, but they are often data-hungry, prone to overfitting in small or heterogeneous clinical cohorts, and vulnerable to various forms of data leakage and flawed model selection in translational EEG studies [13,14,19,54,55,56]. When training data are derived from a single source-reconstruction or connectivity pipeline, models can learn pipeline-specific artifacts rather than generalizable neurophysiological signatures, a limitation which underscores the need for multi-site datasets, harmonized preprocessing and data standards, rigorous cross and external validation, and the exploration of physics-informed or hybrid models that incorporate biophysical priors into machine learning frameworks [12,13,14,19,54,55,56].

Methodological standardization, transparent reporting, and open science are essential for improving reproducibility. Widely used open-source platforms, including EEGLAB, MNE-Python, Brainstorm, and FieldTrip, support transparent and reproducible EEG preprocessing, forward modeling, inverse reconstruction, and connectivity analysis, although their default settings and numerical implementations can differ [12,28,29,68,69]. Standardized data organization and metadata practices, such as the Brain Imaging Data Structure (BIDS) and its EEG extension (EEG-BIDS), facilitate data sharing, pipeline reuse, and independent reanalysis [70]. Reproducible reporting should specify electrode montage and coordinate information, reference strategies, preprocessing and artifact-rejection procedures, head model and conductivity assumptions, source-space definition, inverse method and regularization parameters, source parcellation, connectivity estimation, leakage-correction approaches, graph-construction choices, network-density or thresholding procedures, and machine learning validation strategies.

Community efforts toward shared datasets, open-source code, preregistered analysis plans, benchmark studies, and transparent reporting of methodological choices can help distinguish robust neurobiological findings from pipeline-specific artifacts [12,70]. In parallel, multimodal studies integrating EEG source imaging with MRI, MEG, and fMRI, together with prospective clinical investigations that evaluate source-level and network biomarkers against relevant clinical outcomes, will be essential for translating methodological advances into clinically meaningful diagnostic, prognostic, and therapeutic applications [57,58,64,65,66].

## 9. Conclusions

EEG source localization has developed from a purely technical solution to the inverse problem into a central framework for studying large-scale brain networks, enabling anatomically interpretable analyses of functional and effective connectivity and graph-theoretical organization. Source-space EEG frameworks have enabled a wide range of clinical and cognitive neuroscience applications while also providing features that are informative for machine learning and deep learning. However, their reliability remains tightly coupled to choices in forward modeling, inverse algorithms, preprocessing, connectivity estimation, and graph construction. Addressing persistent challenges—including model uncertainty, source leakage, methodological variability, limited reproducibility of connectivity and graph measures, and the integration of biophysical modeling with data-driven approaches—will require standardized pipelines, multimodal validation, and carefully designed translational studies. Given continued methodological rigor and collaborative validation efforts, source-level EEG and brain network analyses are poised to contribute substantially to the mechanistic understanding of human brain function and the development of robust biomarkers and clinically useful decision-support tools for precision neurology.

## Figures and Tables

**Table 1:** Comparison of representative EEG source reconstruction algorithms. ECD—equivalent current dipole; MNE—minimum-norm estimation; dSPM—dynamic statistical parametric mapping; LORETA—low-resolution electromagnetic tomography; sLORETA—standardized LORETA; eLORETA—exact LORETA; LCMV—linearly constrained minimum variance; DICS—dynamic imaging of coherent sources.

Algorithm	Core Constraint or Prior	Localization and Spatial Characteristics	Robustness to Noise and Computational Demand	Major Limitations	Representative Applications
ECD	A small number of focal dipoles	High accuracy for well-separated focal sources	Moderate computational demand, sensitive to noise, initialization, and model specification	Cannot represent broadly distributed or overlapping activity	Focal epileptiform activity, evoked responses
MNE	Minimum-norm constraint	Distributed estimates with limited spatial resolution and superficial-source bias	Computationally efficient and relatively stable, but sensitive to noise and depth bias	Requires depth weighting or normalization to reduce superficial bias	General cortical mapping, exploratory source imaging
dSPM	Noise-normalized minimum-norm constraint	Distributed estimates with improved statistical interpretability	More robust to noise variation than conventional MNE, with moderate computational demand	Limited spatial resolution and dependence on accurate noise covariance estimation	Task-related activation mapping, group statistical analyses
sLORETA *	Spatial smoothness and standardized current-density estimates	Theoretical zero localization error for a single ideal point source, but intrinsically low spatial resolution	Computationally efficient, relatively stable for distributed patterns	Broad spatial smoothing and limited ability to separate adjacent or correlated sources	Exploratory distributed source mapping, group-level analyses
eLORETA *	Distributed inverse with an exact localization constraint	Theoretical exact localization for a single ideal point source with reduced localization bias relative to sLORETA	Computationally efficient, but spatial resolution remains limited in realistic multi-source settings	Exact localization does not imply high spatial resolution under noisy, multi-source, or correlated-source conditions	Distributed source imaging, source-space connectivity and network studies
Beamforming	Variance-minimization spatial filtering	High spatial specificity, with DICS suited to frequency-specific activity	Moderate computational demand, highly sensitive to covariance estimation and correlated sources	Performance degrades for strongly correlated sources or low signal-to-noise data	Task-related activation mapping, oscillatory and frequency-specific analyses
Bayesian	Explicit priors and posterior inference, often sparsity-promoting	Flexible spatial resolution determined by the selected prior	Often computationally demanding, with noise robustness depending on the prior and model specification	Sensitive to prior selection, hyperparameter tuning, and model assumptions	Advanced source imaging, multifocal or sparse-source inference

* The “exact” or “zero-error” localization properties attributed to sLORETA and eLORETA are theoretical results obtained under specific model assumptions, typically involving idealized point-source conditions, and should not be interpreted as guarantees of high spatial resolution in noisy, multi-source, or correlated-source EEG data.

**Table 2:** Representative functional and effective connectivity measures for source-space EEG analyses. PLV—phase-locking value; PLI—phase-lag index; wPLI—weighted PLI; SNR—signal-to-noise ratio; MVAR—multivariate autoregression; DCM—dynamic causal modeling.

Connectivity Approach	Category	Representative Measures	Main Interpretation	Strengths	Key Limitations and Practical Considerations
Correlation-based	Functional	Pearson correlation, cross-correlation	Linear statistical dependence between source time series	Simple, intuitive, computationally efficient	Highly sensitive to zero-lag leakage, common input, and reference-related confounding
Frequency-domain	Functional	Coherence, imaginary coherency	Frequency-specific linear coupling between source time series	Coherence quantifies frequency-specific coupling; imaginary coherency suppresses zero-lag components and is therefore less sensitive to volume-conduction artifacts	Conventional coherence is susceptible to zero-lag leakage; imaginary coherency may discard genuine near-zero-lag interactions and can have reduced sensitivity at low SNR
Phase-based	Functional	PLV, PLI, wPLI	Consistency of phase relationships across time or trials	Captures phase synchronization; PLI and wPLI reduce sensitivity to zero-lag leakage	Reduced sensitivity at low SNR; PLI and wPLI may underestimate near-zero-lag interactions
Envelope-based	Functional	Amplitude-envelope correlation	Covariation of oscillatory amplitude envelopes	Useful for amplitude coupling and slow network fluctuations	Requires leakage correction, such as orthogonalization; results depend on filtering and correction choices
MVAR-based	Effective	Granger causality, directed transfer function, partial directed coherence	Directed linear interactions based on temporal predictability	Estimates directionality and frequency-specific information flow	Requires adequate data length; sensitive to model order, stationarity, dimensionality, noise, and source leakage
DCM	Effective	Dynamic causal modeling	Directed coupling within a biophysically informed generative model	Supports hypothesis-driven inference and model comparison	Computationally demanding, usually limited to small networks, and highly dependent on model specification and priors

Note: Source-space connectivity analyses reduce, but do not eliminate, the effects of volume conduction and spatial leakage. The choice of estimator, leakage-mitigation method, source parcellation, and network-construction procedure can materially affect connectivity estimates and downstream graph-theoretical measures.

**Table 3:** Interpretation, methodological sensitivity, evidence maturity, and validation priorities of commonly used graph-theoretical metrics in source-level EEG studies.

Metric or Metric Family	Primary Network Interpretation	Main Methodological Sensitivities	Relative Evidence Maturity	Recommended Validation Practice
Global efficiency	Network-wide integration; efficiency of distributed communication	Connectivity estimator, network density, threshold range, weighting and normalization	Relatively mature and widely reported across clinical and cognitive EEG studies; not disease-specific	Confirm stability across justified thresholds and connectivity estimators; interpret with characteristic path length and null-model comparison
Characteristic path length	Global integration; average routing distance across the network	Disconnected graphs, thresholding, density matching, edge weighting	Relatively mature; often complementary to global efficiency	Evaluate together with global efficiency, verify connectedness, and assess robustness across graph-construction choices
Clustering coefficient	Local segregation; neighborhood-level specialization	Thresholding, degree distribution, source parcellation, normalization	Widely used, but findings are heterogeneous across disorders	Compare against appropriate null models and examine convergence with modularity or local efficiency
Modularity	Community structure and functional segregation	Community-detection algorithm, resolution parameter, parcellation, network density	Useful for network reorganization, but cross-study comparability remains limited	Report algorithm and resolution parameter; test robustness across partitions and compare with null models
Centrality and hub measures	Node importance and potential hub vulnerability	Node definition, edge density, weak-edge treatment, thresholding, instability of rank order	Clinically interesting but generally less reproducible	Replicate hub patterns across thresholds, parcellations, and datasets; avoid claims based on isolated regional effects
Rich-club organization	Dense interconnection among highly connected hubs; network core organization	Hub definition, degree-preserving null model, density and threshold selection	Promising but relatively limited and method-sensitive source-level EEG evidence	Use degree-preserving null models; assess stability across justified density thresholds and parcellations, and replicate findings in independent datasets

**Table 4:** Comparison of representative machine learning and deep learning approaches for source-level EEG and brain-network analyses.

Approach	Typical Input	Strengths	Limitations and Computational Considerations	Representative Applications
Classical machine learning	Engineered source, spectral, connectivity, or graph features	Relatively interpretable; effective for moderate sample sizes	Performance depends on feature engineering and may not capture complex nonlinear structure	Clinical classification, biomarker studies, and interpretable prediction
Convolutional neural networks	Source maps, time–frequency representations, and connectivity matrices	Efficient extraction of local spatial and temporal patterns	Require substantial data; sensitive to acquisition variability; limited long-range dependency modeling	EEG decoding, source-map classification, and time–frequency analysis
Recurrent neural networks	Source time series or sequential features	Models temporal dependencies	Sequential training can be computationally demanding and unstable for long sequences	Temporal-state decoding, seizure detection, and event-related analysis
Transformer-based models	Tokenized EEG segments, source time series, and multimodal features	Captures long-range dependencies; supports pretraining and representation learning	High computational and data requirements; risk of overfitting in small cohorts	Pretrained EEG representations, transfer learning, and large-scale decoding
Graph neural networks	Connectivity graphs; brain-network features	Directly incorporates network topology and node features	Sensitive to graph construction and prone to overfitting in small datasets; benefits from regularization and requires careful validation	Brain-network classification, disease prediction, and connectome analysis
Self-supervised or foundational models	Large-scale unlabeled EEG datasets	Learns transferable representations and reduces dependence on manual labels	Requires large diverse pretraining data, domain-shift assessment, and uncertain clinical generalizability	Cross-task transfer, representation learning, and low-label clinical applications

**Table 5:** Clinical maturity, defensible near-term roles, and validation priorities for selected source-level EEG applications.

Clinical Application	Current Evidence Maturity	Most Defensible Current/Near-Term Role	Use Not Yet Supported by Current Evidence	Key Validation Endpoint
Presurgical focal epilepsy	Relatively mature, but center- and protocol-dependent	Multimodal adjunct for convergent localization and presurgical hypothesis generation	Stand-alone determination of surgical target	Concordance with presumed epileptogenic zone, resection site, and postoperative seizure outcome
Dementia and neurodegeneration	Early-to-intermediate; heterogeneous cohorts and endpoints	Longitudinal tracking, subtype/biological stratification, and prognostic research	Stand-alone diagnostic classification	Prospective prediction beyond cognitive testing, structural imaging, and established biomarkers
Traumatic brain injury	Early; variable injury mechanisms, timing, and acquisition conditions	Longitudinal monitoring and risk stratification research	Individual diagnostic or return-to-activity decision-making	Prediction of functional, cognitive, or symptom outcomes beyond clinical assessment and imaging
Schizophrenia and related psychotic disorders	Exploratory; substantial clinical and methodological heterogeneity	Biological stratification and treatment-response research	Diagnostic confirmation or individual-level disease labeling	Replication across sites and incremental prediction of treatment or functional outcomes
Depression and related affective disorders	Exploratory; heterogeneous phenotypes and treatment pathways	Treatment-response prediction and longitudinal monitoring research	Stand-alone diagnostic classification or treatment selection without external validation	Prospective, externally validated improvement in treatment-response prediction beyond clinical measures

## Data Availability

No new data were created or analyzed in this study. Data sharing is not applicable to this article.

## Abbreviations

The following abbreviations are used in this manuscript:

DCM	Dynamic causal modeling
DICS	Dynamic imaging of coherent sources
ECD	Equivalent current dipole
EEG	Electroencephalography
eLORETA	Exact LORETA
fMRI	Functional MRI
LORETA	Low-resolution Electromagnetic Tomography
MEG	Magnetoencephalography
MNE	Minimum-norm estimation
MRI	Magnetic resonance imaging
MVAR	Multivariate autoregressive
PLI	Phase-lag index
ROI	Regions of interest
SAM	Synthetic aperture magnetometry
sLORETA	Standardized LORETA
